# Therapeutic melanoma inhibition by local micelle-mediated cyclic nucleotide repression

**DOI:** 10.1038/s41467-021-26269-w

**Published:** 2021-10-13

**Authors:** Kerstin Johann, Toszka Bohn, Fatemeh Shahneh, Natascha Luther, Alexander Birke, Henriette Jaurich, Mark Helm, Matthias Klein, Verena K. Raker, Tobias Bopp, Matthias Barz, Christian Becker

**Affiliations:** 1 Institute of Organic Chemistry, Johannes Gutenberg University, Mainz, Germany; 2 Institute for Immunology, University Medical Center Mainz, Johannes Gutenberg University, Mainz, Germany; 3 Department of Dermatology, University Medical Center Mainz, Johannes Gutenberg University, Mainz, Germany; 4 Institute of Pharmacy and Biochemistry, Johannes Gutenberg University, Mainz, Germany; 5 Department of Dermatology, University Hospital Münster, Westfälische Wilhelms-University, Münster, Germany; 6 Leiden Academic Center for Drug Research (LACDR), Leiden, Netherlands

**Keywords:** Melanoma, Tumour immunology, Drug delivery

## Abstract

The acidic tumor microenvironment in melanoma drives immune evasion by up-regulating cyclic adenosine monophosphate (cAMP) in tumor-infiltrating monocytes. Here we show that the release of non-toxic concentrations of an adenylate cyclase (AC) inhibitor from poly(sarcosine)-*block-*poly(L-glutamic acid γ-benzyl ester) (polypept(o)id) copolymer micelles restores antitumor immunity. In combination with selective, non-therapeutic regulatory T cell depletion, AC inhibitor micelles achieve a complete remission of established B16-F10-OVA tumors. Single-cell sequencing of melanoma-infiltrating immune cells shows that AC inhibitor micelles reduce the number of anti-inflammatory myeloid cells and checkpoint receptor expression on T cells. AC inhibitor micelles thus represent an immunotherapeutic measure to counteract melanoma immune escape.

## Abstract (web summary)

The acidic tumour microenvironment in melanoma drives immune evasion by cAMP in tumor-infiltrating monocytes. Here, the authors show that the release of an adenylate cyclase inhibitor from micelles restores antitumor immunity and, when combined with regulatory T cell depletion, leads to remission of established B16-F10-OVA tumors.

## Introduction

Cancer cells originate from accumulating genetic alterations and loss of cellular regulatory processes^1^. As a result of genetic changes, tumor cells can express new antigen patterns distinguishing them from normal tissue^2^, and provoking immune responses against them^3,4^. Cancers that are clinically detected have thus escaped or resisted efficient immune attack through immune escape mechanisms^5,6^ and/or immune suppression^7,8^.

A variety of approaches are attempting to increase or restore the effectiveness of immune responses to cancer, most prominently, checkpoint inhibitors^9^. However, dominant tolerance mechanisms in the tumor microenvironment limit the effectiveness of immunotherapeutic approaches. It is, therefore, necessary to decode and reverse tumor immunosuppression in order to improve immune therapies.

Nucleotide signaling molecules serve as universal regulators of metabolism and gene expression in all life forms. Within the immune system, increased intracellular levels of cyclic adenosine monophosphate (cAMP)^10^ repress innate and adaptive immune cell function^11,12^. Regulatory T cells (Treg) take advantage of this effect by suppressing other immune cells through cAMP transmission^13,14^. Possibly due to similar mechanisms, elevated intratumoral cAMP levels in melanoma correlate with melanoma growth, immunosuppression, and metastasis^15–18^. In detailing the role of cAMP in melanoma immunosuppression, we recently observed that human and murine melanomas acidify their microenvironment and thereby upregulate intracellular cAMP levels in tumor-infiltrating monocytes/macrophages, suppressing their antitumor activities^19^.

Taken together, the above observations identify the cAMP pathway as an attractive target for therapeutic intervention in melanoma growth, but its innumerable physiological functions prohibit systemic intervention in the pathway.

In this work, we show that the nucleotide cyclase inhibitor MDL-12,330A^20^ can be incorporated into polypept(o)ide micelles^21–28^ and is continuously released from them locally in melanomas. In contrast to the free drug, MDL-loaded micelles are non-toxic, suppress cAMP formation in tumor tissue and melanoma growth efficiently. Suppression of tumor growth by micelle-mediated nucleotide cyclase inhibition requires a complete immune system and is associated with quantitative and functional changes in multiple intratumoral immune cell populations. In combination with occasional non-protective depletion of Treg, micelle-mediated cAMP suppression leads to complete tumor remission.

## Results and discussion

### Synthesis and characterization of polypept(o)ide micelles

Amphiphilic block copolypept(o)ides (pSar_195_-*block*-pGlu(OBn)_25_) were synthesized by sequential nucleophilic ring-opening polymerization of the corresponding *α*-amino acid *N*-carboxy anhydrides (NCA) according to Birke et al. ^23^. The block copolymer displayed a molecular weight of 32.5 kg/mol and dispersity of 1.15 marking controlled polymerization (Supplementary Fig. 1). Block co(polypept(o)ides) have low cellular toxicity and do not cause immune-stimulatory effects^22^.

**Fig. 1:**
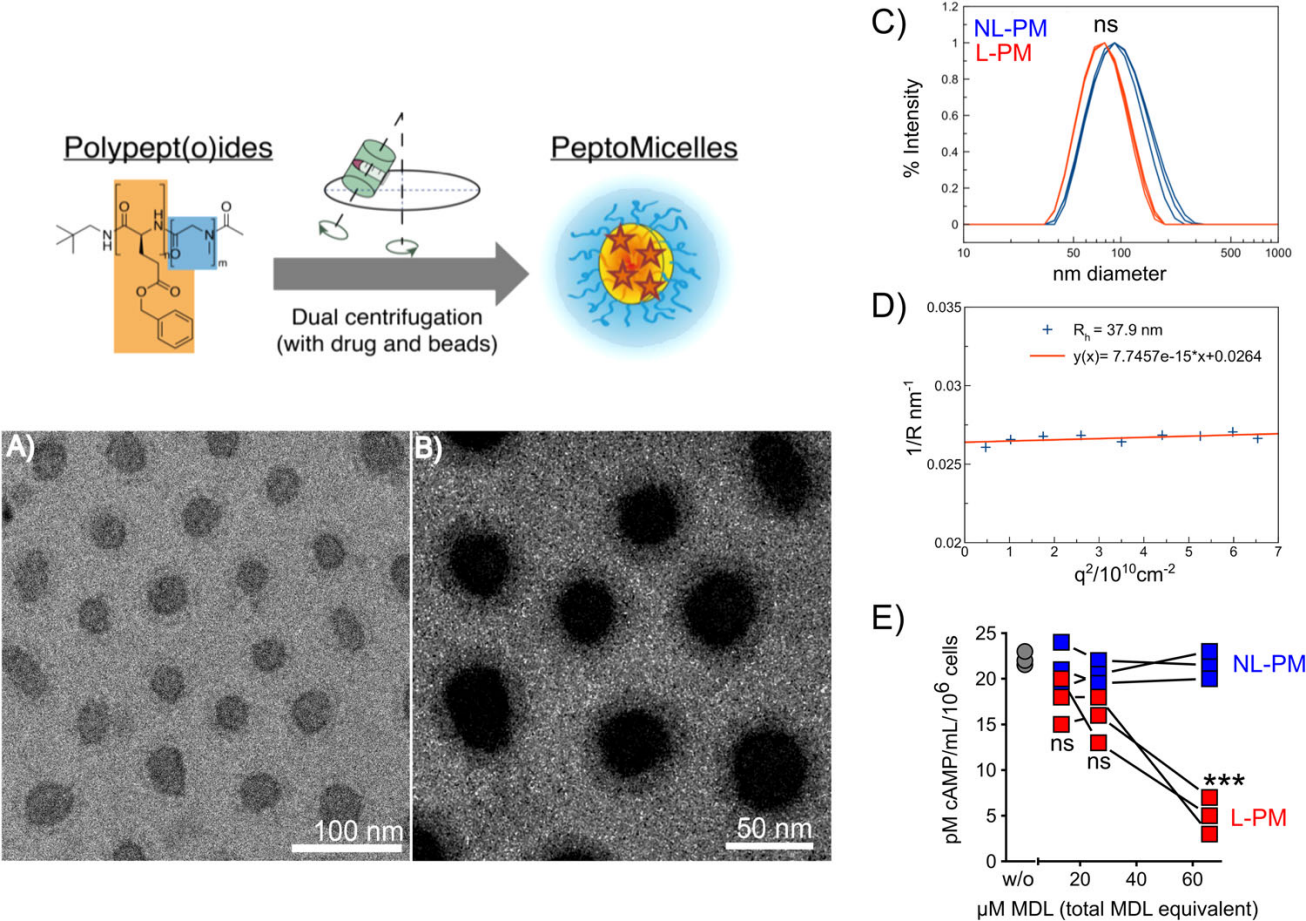
MDL-loaded polypept(o)ide micelle properties. Schematic representation of polypept(o)ide micelle synthesis and inhibitor loading. **A** Cryo TEM images of MDL-loaded micelles at 1 mg/mL and **B** a magnification with optimized contrast after applying a Gaussian blurr effect with ImageJ to increase the visibility of the corona layer. **C** Single-angle DLS measurement of unfiltered solutions of empty (NL-PM, blue) and loaded (L-PM, red) micelles directly after centrifugation (*n* = 3; diameter differences were statistically nonsignificant *P* = 0.69 (unpaired Mann–Whitney test, two-sided, 0.95 confidence interval, significance level 0.05. **D** Multi-angle DLS data of L-PM to verify and back up zetasizer data. **E** Dose-dependent repression of intracellular cAMP levels in murine B16 cells, 4 h after incubation with different amounts of L-PM or NL-PM micelles. Results shown as mean ± SEM, *n* = 3 per treatment/dose, ***P = 0.003 (66 µM) (unpaired *t*-test with Holm–Šídák-corrected *P* values, alpha = 0.05, 0.95 confidence interval). ns = nonsignificant. Each image and graph representative of *n* = 3 independent experiments.

MDL-12.330A (MDL) inhibits all adenylate cyclases irreversibly^29^. To prepare MDL-loaded micelles, amphiphilic block copolypept(o)ides were subjected to dual centrifugation (cartoon Fig. 1)^30^ in the presence of one equivalent of the drug and subsequently purified by spin filtration. The well-scalable approach allows for fast preparation of loaded micelles under high shear conditions, without the use of organic solvents, sterile in a sealed vial. The resulting micelles contained a reproducible 9% drug load as determined by RP-HPLC (Supplementary Figs. 2 and 3); 60% of formulated MDL ended up in the final micellar formulation, equivalent to 60% loading efficiency. By comparison, drug loading by solvent exchange only leads to a drug load of <1%. Lyophilized or 50–150 mg/mL aqueous solutions of drug-loaded micelles stored at 4 °C for several months without loss of activity.

MDL-loaded micelles (L-PM) displayed an average diameter of around 76 nm while the non-loaded micelles (NL-PM) showed a hydrodynamic diameter of 98 nm (Fig. 1A–D). This indicates that the drug enhances phase separation of the amphiphilic block copolymer, suggesting an ordered micellar structure in its presence. Cryogenic transmission electron microscopy (cryo-TEM) analysis revealed spherical micelles with an average diameter of 61 ± 12 nm, respectively. It also confirmed a core with higher electron scattering contrast, surrounded by a less dense corona, indicating that the hydrophobic segment and the drug form a compact core while the hydrophilic polysarcosine block forms the corona. Multi-angle dynamic light scattering (DLS) data were in line with both zeta sizer and cryo-TEM results, as expected for narrowly distributed spherical micelles. Table 1 summarizes the micelle characteristics.

**Table 1:** Characterization of MDL-loaded and non-loaded polypept(o)ide micelles.

	Diameter (nm)		*Z*-average (nm)^c^	PDI^c^	C total (mg/mL)^d^	C MDL (mg/mL)^e^	Drug load (%)
	Cryo-TEM^a^	MA DLS^b^					
L-PM	61 ± 12	75.8	72.1 ± 0.7	0.07 ± 0.02	120	11.15	9.29
NL-PM	n.d.	n.d.	97.8 ± 1.8	0.25 ± 0.02	64	0	0

Errors indicate one std. deviation of multiple measurements (if applicable).

^a^As determined by measuring two perpendicular axes of 33 particles with ImageJ.

^b^As determined by multi-angle DLS (MA DLS).

^c^As determined by single-angle Dynamic Light Scattering (SA DLS).

^d^As determined by lyophilization of a portion of injected solutions.

^e^As determined by HPLC.

B16 melanoma cells have constitutively elevated intracellular cAMP levels^19^ allowing inhibitor-mediated decreases to be easily determined. To test the ability of MDL-loaded micelles to release the incorporated adenylate cyclase inhibitor, we incubated B16 melanoma cells with different amounts of MDL-loaded (L-PM) or non-loaded (NL-PM) polypept(o)ide micelles and subsequently determined their intracellular cAMP level. L-PM, but not NL-PM, reduced the intracellular cAMP level in cultured B16 melanoma cells in a dose-dependent manner over 75% (Fig. 1E), proving an efficient inhibitor release.

### Peritumoral retention of polypept(o)ide micelles

MDL-loaded micelles were designed to continuously release the inhibitor locally at the tumor tissue and are excreted or metabolized by the body upon enzymatic decay. Primary cutaneous melanomas are easily accessible and thus allow for testing local treatment approaches.

**Fig. 2:**
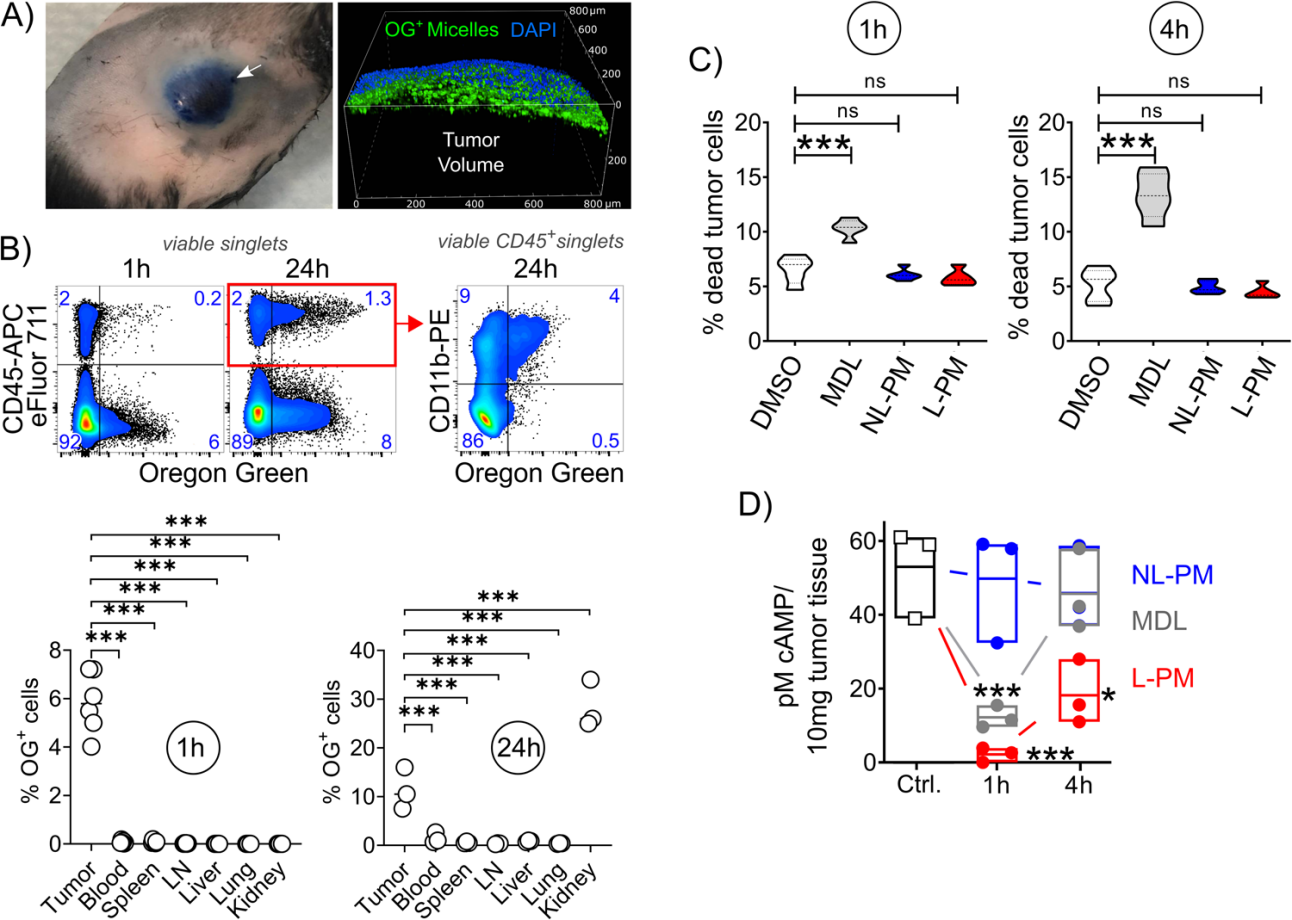
Effect and distribution of peritumorally injected MDL-loaded polymer micelles. **A** Peritumoral injection. Left: Image of a mouse with B16 melanoma after peritumoral injection of Evans Blue, injection site indicated by the arrow, fur removed for better visibility; right: Confocal microscopic image of the tumor margin after peritumoral injection of Oregon Green (OG)-labeled micelles. **B** Frequency of OG-labeled cells in different organs 1 and 24 h after peritumoral injection of OG-labeled L-PM micelles at day 14 B16-OVA melanomas. Representative flow cytometric plots, numbers represent cell frequencies in gates. LN lymph nodes (*n* = 6 (1 h) and *n* = 3 (24 h) per organ, all comparisons ***P < 0.001 (parametric one-way ANOVA, with Holm–Šídák’s multiple comparisons test, alpha = 0.05, 0.95 confidence interval). **C** Micelles (NL-PM, blue; L-PM, red), free inhibitor (MDL, gray), and solvent (DMSO, white) toxicity in tumor tissue 1 and 4 h after peritumoral injection at day 14 melanomas. Violin plots show median, interquartile range, minimum and maximum (truncated above and below max and min values) of dead CD45^neg^CD29^+^ tumor cells, *n* = 5 per treatment, ***P = 0.00099 (1 h) ***P = 0.00099 (4 h) ns = nonsignificant (parametric One-way ANOVA, with Holm–Šídák’s multiple comparisons test, alpha = 0.05, 0.95 confidence interval). **D** Intracellular cAMP levels in day 14 B16-OVA melanoma tissue after peritumoral injection of 10 µg MDL dissolved in DMSO, L-PM (10 µg MDL) or NL-PM (same amount of particle substance as L-PM). Data shown as mean ± SEM, *n* = 3 per treatment, *P = 0.006 (NL-*P*M vs. L-PM, 4 h), ***P = 0.00099 (NL-PM vs. L-PM, 1 h), ***P = 0.00099 (MDL vs. NL-PM, 1 h) (parametric one-way ANOVA, with Holm–Šídák’s multiple comparisons test, alpha = 0.05, 0.95 confidence interval). All figures and graphs are representative of three independent experiments each.

The retention and penetration of nanoparticles into tissues are determined by their size, shape, and surface chemistry, but cannot be predicted from their chemical properties^31^. To probe melanoma retention of polypept(o)ide micelles, we injected Oregon-Green-labeled L-PM micelles peritumorally at B16F10-OVA melanomas grown subcutaneously for 14 days in C57BL/6 mice and analyzed the dye distribution to various tissues 1 and 24 h later. The particles were injected peritumorally, not intratumorally, to surround the tumor mass with the injected solution without causing tissue damage (Fig. 2A). Intravital microscopy showed that peritumorally injected micelles remained in the connective tissue around the tumor and did not enter the central tumor mass (Fig. 2A and Supplementary Movie 1). Within the first hour after peritumoral injection, the particle dye remained almost exclusively in the tumor tissue and was absorbed by CD45^+^ immune and non-hematopoietic CD45^neg^ cells (Fig. 2B and Supplementary Fig. 4A). Among immune cells, micelles interacted selectively with myeloid (CD11b^+^) cells (Fig. 2B). Only after a longer period of time (24 h) particle-bound fluorescence was also found in the kidney (Fig. 2B) and there mainly in non-hematopoietic cells. Based on the hydrodynamic diameters of individual polymers or polymer fragments below the renal threshold (*R*_h_ = 1.5–3 nm), the latter observation suggests renal clearance^32^. L-PM was used throughout for the retention figures. Micelles loaded with the FDA-approved near-infrared (NIR) fluorescent agent Indocyanine Green (ICG) also confirmed local retention after peritumoral injection (Supplementary Fig. 5).

To verify the safety of micelle-mediated cAMP inhibitor administration, we also determined the frequency of dead cells in peritumorally injected day 14 melanomas. For comparison, we injected day 14 melanoma with NL-PM, L-PM, the free drug, or its solvent (DMSO). Combined annexin V/7-amino-actinomycin (7-AAD) and cell surface marker staining at 1 and 4 h post-injection revealed some dead (CD45^neg^CD29^+^) melanoma cells after injection of a high dose of free drug (10 µg). In contrast, a nominally equal micellar dose remained non-toxic (Fig. 2C and Supplementary Fig. 4B). This observation shows that the use of micelles avoids the toxic effects of the inhibitor.

### cAMP repression in melanoma by MDL-loaded polypept(o)ide-micelles

Peritumoral retention of particle fluorescence upon local administration indicated that the particle design supports local drug release. However, the measurement of particle-bound Oregon Green does not document the fate of the cAMP inhibitor MDL. To determine the inhibitor release from MDL-loaded micelles in vivo, we injected the free drug and micelles peritumorally into day 14 melanomas and subsequently determined intratumoral cAMP levels at different time points. Both the free drug and inhibitor-loaded micelles reduced intracellular cAMP levels in B16 melanomas within the first hour after injection. However, while with the free drug, intratumoral cAMP levels returned to the level of untreated tumors after 4 h, they remained reduced with inhibitor-loaded micelles (Fig. 2D), which shows that the release of MDL from micelles extends its local availability.

### Melanoma growth inhibition with MDL-loaded polypept(o)ide micelles

**Fig. 3:**
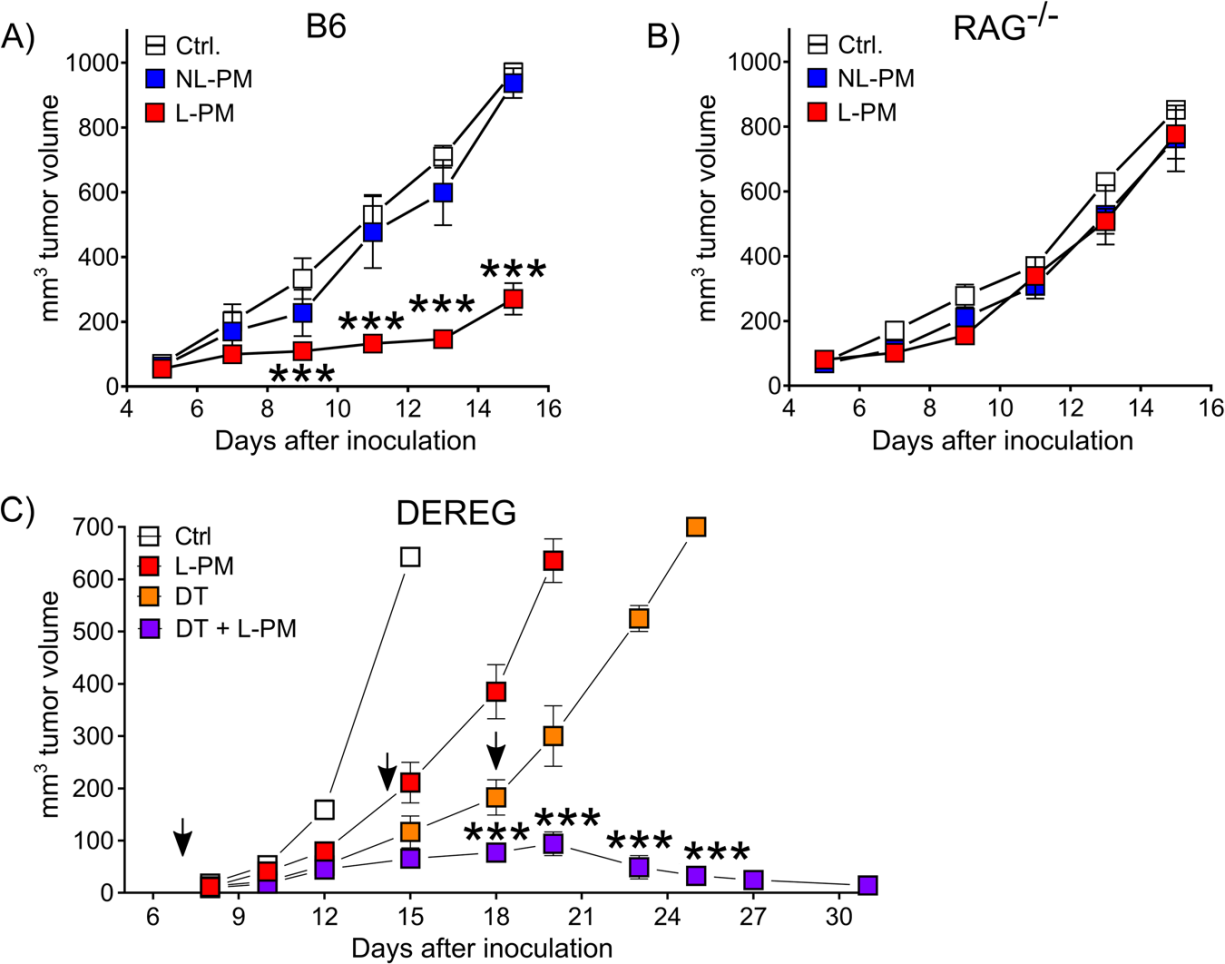
MDL-loaded micelles repress melanoma growth. Inhibition of B16 melanoma growth by peritumorally injected MDL-loaded micelles in **A** C57BL/6 mice, **B** B6.RAG^−/−^ and **C** DEREG mice. Mice were inoculated s.c. with B16-OVA tumor cells and received peritumoral micelle injections every other day starting from day 4 after inoculation. **A** Representative results of three independent experiments shown as mean ± SEM, Ctrl (white) *n* = 5, L-PM (red) *n* = 6, NL-PM (blue) *n* = 5, ***P = 0.000988 (day 9), ***P = 0.000989 (day 11), ***P = 0.00099 (day 13), ***P = 0.00098 (day 15) (parametric unpaired two-sided *t*-test with Holm–Šídák-corrected *P* values, alpha = 0.05, 0.95 confidence interval). **B** Representative results of three independent experiments shown as mean ± SEM, *n* = 5 per group, *P*-values did not reach statistical significance (parametric unpaired two-sided *t*-test with Holm–Šídák-corrected *P* values. **C** Some mice received additional diphtheria toxin (DT) injections at days 7, 14, and 18 (arrows). Representative results of two independent experiments shown as mean ± SEM, Ctrl (white) *n* = 5, L-PM (red) *n* = 4, DT (orange) *n* = 4, DT+L-PM (violet) *n* = 5, Ctrl vs. L-PM: ***P = 0.00098 (day 15); Ctrl vs. DT: **P = 0.007 (day 12), ***P = 0.00098 (day 15); DT vs. DT+L-PM: ***'P = 0.000998 (day 18), ***P=0.000997 (day 20), ***P = 0.00098 (day 23), ***P = 0.00099 (day 25) (parametric unpaired two-sided *t*-test with Holm–Šídák-corrected *P* values, alpha = 0.05, 0.95 confidence interval).

To ensure a continuous inhibition of cAMP formation in the tumor tissue, we injected the micellar drug formulations every second day, beginning once the tumor became palpable (around day 4 after inoculation). Mice injected with MDL-loaded micelles receiving 200 µM total drug content (based on HPLC quantification, see Supplementary figures) showed significantly reduced melanoma growth (Fig. 3A). Tumor growth repression by MDL-loaded micelles was dose-dependent as suggested by the non-effectivity of a 10-fold reduced micellar inhibitor dose (Supplementary Fig. 6A) and more effective than the free drug (Supplementary Fig. 6B). Suppression of tumor growth by cAMP repression had no discernible side effects, be it cellular toxicity (Fig. 2C) or discomfort of the animals. We also performed experiments in which we started the micelle injection on day 6 after tumor inoculation. L-PM suppressed tumor growth in a delayed manner in this regimen (Supplementary Fig. 5C), demonstrating a therapeutic effect in more established tumors.

In order to narrow down possible target cells, we conducted experiments in RAG knockout mice lacking adaptive immune cells. In contrast to immunocompetent mice, inhibitor-loaded micelles could not influence the growth of B16 melanoma in RAG-deficient mice (Fig. 3B). Thus, in accordance with the fluorescence distribution of OG-labeled micelles, the immune infiltrate appears to be essential for the anti-tumor effect observed.

### Melanoma rejection through combined Treg/cAMP interference

The inability to repress melanoma growth through cAMP interference in RAG-deficient mice revealed that adaptive immune cells play a crucial role in the therapeutic effect. Among the adaptive immune cells, Treg are distinguished by their elevated intracellular cAMP levels and their ability to regulate other immune cells by cAMP transmission via gap junctions^13^. In line, Treg are characterized by FOXP3-induced decreased PDE3B expression^33^ and increased adenylate cyclase 9 (AC9) activity^34^, and, non-functional Treg in *Foxp3*-mutant scurfy mice harbor significantly reduced cytosolic cAMP levels^35^. Pharmacological inhibition of cAMP formation by either AC inhibition, application of a cAMP-specific antagonist, or PDE overexpression abrogates murine and human Treg suppression^13,36–39^. Inversely, blockade of cAMP degradation by PDE inhibition improves Treg-mediated suppression^40^. Moreover, IFN-α, used in adjuvant melanoma therapy, was found to block Treg suppressive activity by interfering with cAMP production^14^.

Selective depletion of Foxp3^+^ Treg constitutes a highly effective strategy to improve the outcome of anti-tumor treatments^41^ and reagents for their removal in humans are under evaluation^42,43^. We, therefore, asked whether reducing Treg frequencies could synergize with micellar cAMP repression and combined micellar cAMP repression with punctual, non-protective Treg-depletion in (depletion of regulatory T cell (DEREG) BAC transgenic) mice. While occasional and incomplete Treg depletion slowed tumor growth, combined cAMP repression/Treg deletion achieved complete tumor rejection (Fig. 3C). This shows that cAMP repression as a means of immune activation combines well with other immunostimulatory or suppression-overriding treatments.

### Micellar peritumoral cAMP suppression prevents tumor immune cell dysfunctionality

**Fig. 4:**
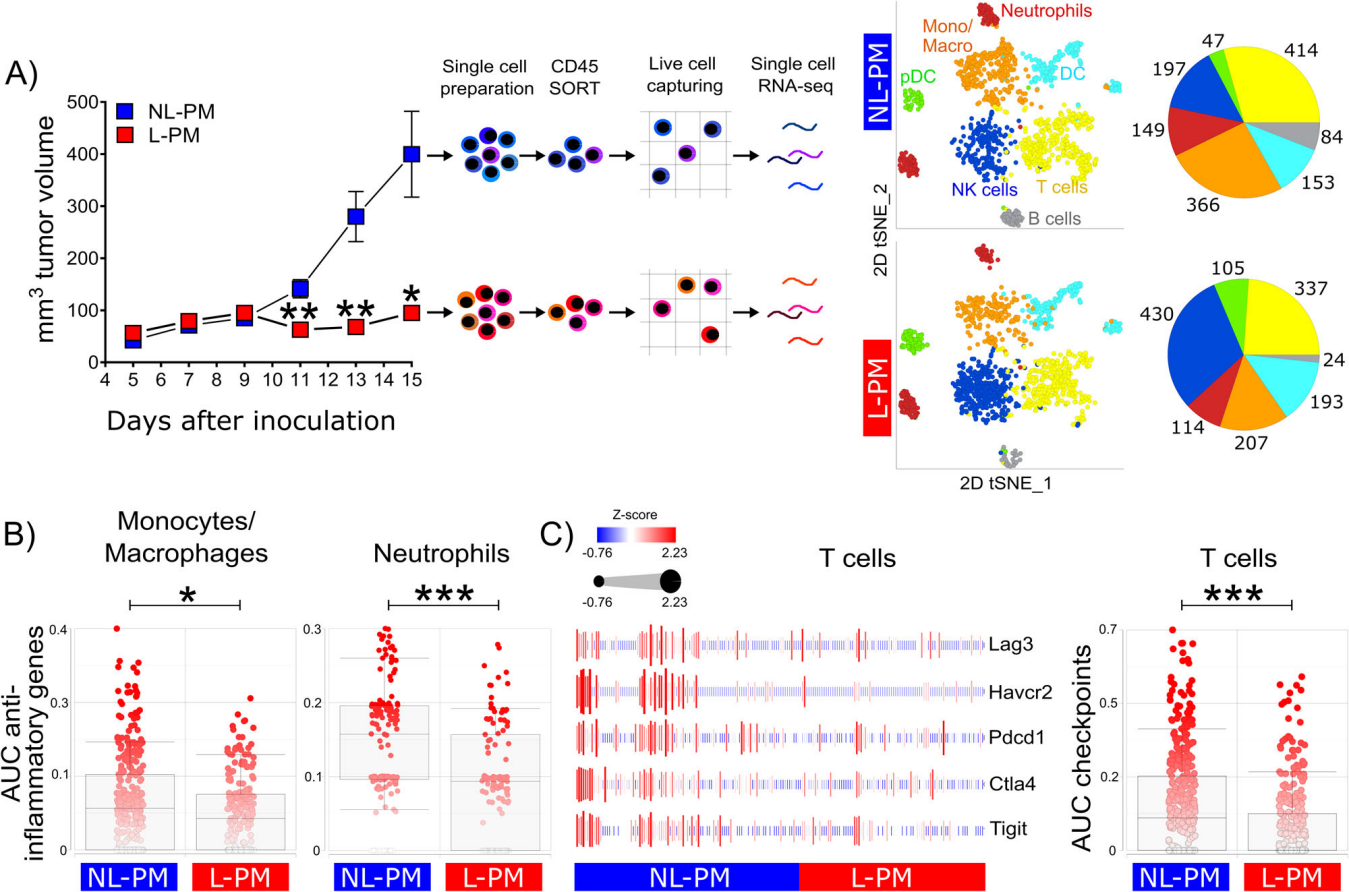
Micellar peritumoral cAMP suppression prevents tumor immune cell dysfunctionality. **A** Tumor volumes, experimental workflow, and unsupervised cell clustering. C57BL/6 mice were subcutaneously inoculated with B16-OVA tumor cells and received peritumoral micelle injections every other day starting from day 4 after inoculation. Tumor volumes shown as mean ± SEM, *n* = 10 per group, NL-PM (blue) vs. L-PM (red): **P = 0.0079 (day 11), **P = 0.0084 (day 13), *P = 0.0196 (day15); (parametric unpaired two-sided *t*-test with Holm–Šídák-corrected *P* values, alpha = 0.05, 0.95 confidence interval). At day 15, CD45^+^ single cells were isolated from tumor tissue and subjected to single-cell transcriptome profiling. 2D tSNE plots of color-coded cell populations and pie charts with numbers of clustered cells (monocytes/macrophages, orange; neutrophils, red; dendritic cells (DC), light blue; plasmacytoid DC, green; natural killer (NK) cells, dark blue; T cells, yellow; B cells, gray). **B** AUC scores using an anti-inflammatory input gene set (*Ptgs2*, *Vegfa*, *Egfr*, *Arg1*, *Ccl22*, *Ccl17*, *Il10,* and *Il12a*) on monocytes/macrophages and neutrophils treated with NL-PM or L-PM. *P = 0.024, ***P = 0.001 (non-parametric unpaired two-sided Mann–Whitney test). **C** Heatmap showing *z*-scored average expression of indicated exhaustion marker genes in T cells treated with NL-PM or L-PM (left) and AUC score using the heatmap’s exhaustion marker gene set as input on NL-PM or L-PM treated T cells (right). ***P = 0.00099 (non-parametric unpaired two-sided Mann–Whitney test, alpha = 0.05, 0.95 confidence interval). Data are shown as box and whiskers plots. Centerlines represent the median, the edges of the box indicate the 25th and 75th percentiles and the whiskers represent the 10th and 90th percentiles. Single-cell RNA-Seq data are accessible via NCBI Gene Expression Omnibus (GEO) database accession number: GSE166028 [https://www.ncbi.nlm.nih.gov/geo/query/acc.cgi?acc=GSE166028].

Elevated intracellular cAMP levels lower innate and adaptive immune functions^11,12^ and induce a non-inflammatory phenotype in melanoma-infiltrating monocytes/macrophages^19^. In order to understand how the micellar cAMP inhibition enables efficient anti-tumor immunity, we performed single-cell level mRNA expression profiling of all CD45^+^ tumor-infiltrating immune cells in micelle-treated melanomas. After data processing, quality control, and random downsampling, 2820 CD45^+^ tumor-infiltrating cells per sample were kept for downstream analyses (see the “Methods” section). Unsupervised clustering on the high-dimensional space and subsequent classification employing IMMGEN databrowser (www.immgen.org) revealed the presence of B cells, dendritic cells (DC), plasmacytoid dendritic cells (pDC), macrophages/monocytes (Mono/Macro), neutrophils, NK cells, and T cells (Fig. 4A). Further analyses revealed quantitative changes in several immune cell populations. While B cell, macrophage/monocyte, and neutrophil granulocyte numbers were reduced, a concomitantly strong increase in cells expressing cytotoxic NK cell-associated genes as well as genes associated with pDC could be observed (Fig. 4A).

Next to quantitative changes, especially qualitative changes might contribute to the strong anti-tumor effect observed upon peritumoral administration of L-PM. Analyzing the expression of genes associated with an immunosuppressive or alternatively activated state in myeloid cells demonstrated downregulation of *Ptgs2* (Prostaglandin-Endoperoxide Synthase 2), *Vegfa* (Vascular endothelial growth factor A), *Egfr* (Epidermal growth factor receptor), *Arg1* (Arginase 1), *Ccl22* (C–C Motif Chemokine Ligand 22), *Ccl17* (C–C Motif Chemokine Ligand 17), *Il1rn* (Interleukin 1 Receptor Antagonist), *Il10* (Interleukin 10) and *Il12a* (Interleukin 12A) expression in the macrophage/monocyte cluster as well as in the neutrophil granulocyte cluster (Fig. 4B and Supplementary Fig. 7).

Immunosuppressive myeloid cells in the tumor contribute to T cells adopting a dysfunctional state characterized by upregulation of several immune checkpoints^44^ Of note, peritumoral administration of L-PM resulted in a strong decrease in expression of *Lag3* (LAG3), *Ctla4* (CTLA-4), *Pdcd1* (PD-1) and *Havcr2* (TIM3) and, somewhat less strongly, *Tigit* (T Cell Immunoreceptor with Ig and ITIM domains) by intratumoral T cells (Fig. 4C). These data support the notion that particle-mediated suppression of cAMP formation counteracts dysfunctional differentiation of T cells in tumors.

Taken together, our data show that particle-mediated suppression of cAMP formation helps the immune system fight melanoma by preserving the functionality of immune cells in melanoma tissue.

In summary, we provide preclinical evidence that local cAMP repression with inhibitor-loaded polymeric micelles reduces melanoma growth. We show that it is safe, relies on immune activation, and provides a suitable way for the development of optimized local melanoma immunotherapies.

## Methods

### Polymer synthesis

The synthesis of Glu(OBn) NCA, Sar NCA, and the pGlu(OBn)_25_-*b*-_p_Sar_195_ polymer was performed according to Birke et al. ^23^. First, both NCAs have been synthesized using the Fuchs-Farthing method. The polymerization of Glu(OBn) NCA was always carried out at 0 °C in abs. DMF to prevent a loss of end groups by pyroglutamate formation. The chain lengths were set to be 25 by the monomer to initiator ratio.

For the polymerization, 527.9 mg of Glu(OBn) NCA (1.98 mmol) were transferred under nitrogen counter flow into a pre-dried Schlenk-tube equipped with a stir-bar and again dried in high vacuum for 1 h. The NCA was then dissolved in 4 mL of dry DMF. 13.86 µL of neopentylamine were added to 1.5 mL of dry DMF in a glass vial flooded with argon and mixed. For initiation, 1 mL of the prepared solution was directly added to the Schlenk-tube via syringe (yielding a total of 5 mL with ~100 mg/mL in respect to the monomer). The solution was stirred at 0 °C and kept at a constant pressure of 1.25 bar of dry nitrogen via the Schlenk-line to prevent impurities from entering the reaction vessel while allowing CO_2_ to escape. Completion of the reaction was confirmed by IR spectroscopy (disappearance of the NCA peaks (1853 and 1786 cm^−1^)). After completion of the reaction, a solution containing 1754.0 mg of sarcosine NCA in 15 mL dry DMF was added via syringe and allowed to warm to room temperature. After complete consumption of Sar NCA as verified by IR spectroscopy, 80% of the solution were treated with 10 equivalents of acetic acid anhydride and 20 equivalents of triethylamine per chain end and subsequent stirring overnight at room temperature. The uncapped 20% portion was directly precipitated to cold ether and centrifuged (2540×*g* at 4 °C for 15 min) for further functionalization; the end-capped polymer was precipitated the next day, respectively. After discarding the liquid fraction, ether was added again and the polymer was re-suspended in a sonic bath. The suspension was centrifuged again and the procedure was repeated twice. After purification by the re-suspension steps, the polymer was dispersed in water and lyophilized, yielding 1054.1 mg (97% yield) of a colorless powder.

^**1**^**H NMR:** pGlu(OBn)_25_*-b-*pSar_195_ (400 MHz, DMSO−*d*_6_): *δ* [ppm] = 8.70–7.60 (22H, br, –**NH**–CO–CH–), 7.56–6.90 (131H, br, –C_6_H_5_), 5.25–4.70 (50H, br, –O–**CH**_**2**_–C_6_H_5_), 4.65–3.65 (412H, br, –NCH_3_–**CH**_**2**_–CO– and –CO–**CH**–NH–), 3.15–2.60 (595H, br, –N**CH**_**3**_, 1.85–0.94 (67H, br, –**CH**_**2**_**–CH**_**2**_–), 0.84 (9H, s, –C(**CH**_**3**_)_3_) GPC as performed in hexafluoroisopropanol (HFIP) using a PMMA calibration: *M*_n_ = 32.5 kg/mol, dispersity index Đ = 1.15.

Labeling of block copolypept(o)ides with Oregon Green 488 succinimidyl ester (OG488-NHS): For end-group functionalization of block copolypept(o)ides with Oregon Green (OG488-NHS), 60.0 mg (of the non-acetylated) polymer were dissolved in 1 mL of DMF and 1.25 equivalents of OG488-NHS, dissolved in 0.5 mL of DMSO, were added. The solution was stirred for 16 h at 0 °C and 8 h at 40 °C. Afterward, the polymer was precipitated to cold ether and centrifuged (3215×*g* at 4 °C for 15 min). After discarding the liquid fraction, the polymer was suspended in water and lyophilized. A total of 20 mg of the lyophilized polymer was dissolved in 50/50 THF/water and purified by preparative reverse-phase HPLC. The THF was removed using a rotary evaporator and the residue lyophilized, yielding 10.02 mg (50%) of purified, fluorescently labeled polymer. Absorption at 496 nm quantified using the Lambert–Beer-Law suggested 53% end-group labeled unimers as determined by UV–Vis spectrophotometry (V-630; JASCO corp.; for details and calculation see SI).

### Micelles

Micellar formulations of MDL were prepared by dual centrifugation upon vigorously mixing the block copolypept(o)ide and the drug. The acetyl-capped poly(*γ*-benzyl-l-glutamatic acid)_25_-*block*-polysarcosine_195_ was dissolved in chloroform, filtered with 200 nm PTFE Filters (CHROMAFIL^®^ O-20/15 MS), and dried inside 750 µL vials overnight using an Eppendorf Concentrator Plus vacuum centrifuge.

To the 45 mg of previously transferred polymer, 5 mg of MDL, and 175 mg of ceramic beads (SiLiBeads ZY 0.3–0.4 kindly provided by Sigmund Lindner, Warmesteinach, Germany) were added. 450 µL of Millipore water were added and the polymer was allowed to swell for one hour. 0.53 mg sodium hydroxide dissolved in 50 µL of Millipore water (1.25 eq of NaOH per MDL) were added directly prior to centrifugation, which was performed for 20 min at 1048×*g* with a dual centrifuge (Rotanta 400 DC prototype which kindly provided by Andreas Hettich GmbH, Tuttlingen, Germany). The centrifuge was operated with custom 3D-printed sample holder fittings.

After centrifugation, the turbid solution was separated from the beads with an Eppendorf pipette and the vial was rinsed twice with Millipore water. The solutions were transferred to a spin filter and free MDL was separated by spin filtration using Amicon^®^ Ultra—2 mL Centrifugal Filters (Ultracel^®^ 100 K) at 1429×*g*. Samples of the filtrate were subjected to HPLC while a portion of the purified micellar solution was lyophilized to obtain the total mass concentration.

ICG-loaded micelles were prepared by dual centrifugation, analogous to MDL-loaded micelles, but without the addition of sodium hydroxide.

### Characterization of micelles

400 MHz ^1^H NMR spectra were recorded on a Bruker Avance-II 400. Spectra were recorded at room temperature and analyzed with MestReNova 10.0 software. Degrees of polymerization was calculated by comparing the integral of the neopentylamine initiator peak and the integrals of benzylic methylene groups of the protection group for the protected amino acids and the average of integrals of the methyl groups and *α*-protons for sarcosine in the 400 MHz ^1^H NMR spectra.

HFIP gel permeation chromatography (GPC) was performed with HFIP containing 3 g/L potassium trifluoroacetate as eluent at 40 °C. The columns were packed with modified silica (PFG columns; particle size: 7 µm, porosity: 100 and 1000 Å). The polymer was detected with a refractive index (RI) detector (G 1362A RID, JASCO) and a UV/Vis detector (UV-2075 Plus, JASCO). Molecular weights were calculated using a calibration performed with PMMA standards (Polymer Standards Services GmbH, Mainz, Germany) and toluene as an internal standard. Elution diagrams were analyzed using the WinGPC UniChrome 8.00 (Build 994) software from Polymer Standards Services.

Attenuated total reflectance Fourier-transformed infrared (ATR-FTIR) spectroscopy was performed on an FT/IR-4100 (JASCO Corporation) with an ATR sampling accessory (MIRacle^TM^, Pike Technologies) and analyzed using Spectra Manager Version 2.0 (JASCO Corporation).

DLS at a static scattering angle of 173° was performed on a Malvern Zetasizer Nano ZS. 1 µL of the MDL micelle solution was diluted to 1 mL MilliPore-water in disposable polystyrene cuvettes. After equilibration to 25 °C three measurements with 12 runs each were performed. The RI, viscosity, and RI of the particle were set to 1.330, 0.8872 cP, and 1.45, respectively. The absorption of the particle was set to 0.01, while the attenuator and the measurement position were optimized by the instrument.

For multi-angle DLS, samples at a concentration of 1 mg/mL were filtered with 450 nm GHP Filters (Acrodisc^®^ PSF Syringe Filter, PALL Life Sciences) into dust-free cylindrical scattering cells (Suprasil, 20 mm diameter, Hellma, Mühlheim, Germany). The DLS measurements were performed using a Uniphase He/Ne Laser (*λ* = 632.8 nm, 25 mW), an ALV-SP125 goniometer, an ALV/High QE APD Avalanche photodiode with fiber optical detection, an ALV 5000/E/PCI correlator, and a Lauda RC-6 thermostat unit at 20 °C. Angular-dependent measurements were carried out in the range of 30°−150° at steps of 15°. For data evaluation experimental intensity correlation functions were transformed into amplitude correlation functions applying the Siegert relation extended to include negative values after baseline subtraction by calculation *g*_1_(*t*) = SIGN (*G*_2_(*t*))·SQRT(ABS(*G*_2_(*t*)−*A*)/*A*), with *A* the measured baseline and *G*_2_(*t*) the experimental intensity correlation function. All field correlation functions were fitted by a sum of two exponentials *g*_1_(*t*) = *a*·exp(−*t*/*b*)+*c*·exp(−*t*/*d*) to take polydispersity into account. Average apparent diffusion coefficients *D*_app_ were obtained by applying *q*^2^·*D*_app_ = (*a*−*b*−1 + *c*·*d*−1)/(*a* + *c*) resulting in an angular-dependent diffusion coefficient or reciprocal hydrodynamic radius 〈1/*Rh*〉_app_. The *z*-average hydrodynamic radii *R*_h_ were obtained by extrapolation of 〈1/*R*_h_〉_app_ to *q* = 0. The normalized second cumulant *μ*_2_ was calculated using a cumulant fit at 90°.

### Quantification of micelle loading

HPLC was performed with an Agilent 1100 system equipped with a quad pump, a diode array detector recording at 254 nm, a YMC Triart C18 RP column, and running the following acetonitrile (ACN) gradient with a constant concentration of 20 mM triethylammonium acetate (TEAA; constant 2% of an 1 M TEAA solution in water): 0 min: 1% ACN, 1 min: 1% ACN, 25 min: 98% ACN, 30 min: 1% ACN.

The concentration of MDL in the micellar formulation was determined indirectly by subtracting the free MDL quantified in the filtrate (as determined by HPLC) from the amount added initially to the preparation. The intensity of the filtrate MDL peak relative to a calibration curve obtained by injection of 0.25–7.5 µg MDL (0.25, 0.5, 0,75, 1, 1.5, 2, 3, 4, 5, and 7.5 µg) yielded the amount of separated MDL (*c*_HPLC_*V_filtrate_). 50 µL of the micelle-enriched solution was lyophilized to yield the total mass of micelles (*m* (polymer + MDL)).

Drug load was calculated using the concentration of MDL divided by the mass of total contents as determined by lyophilization. The calculated drug load for the injected formulation was determined to be 9.2%.

1 $${{{{{\rm{drug}}}}}}\; {{{{{\rm{load}}}}}}\left(\frac{w}{w}\right)=\frac{{m}({{{{{\rm{total}}}}}}\; {{{{{\rm{MDL}}}}}})-{m}({{{{{\rm{free}}}}}}\,{{{{{\rm{MDL}}}}}})}{{m}({{{{{\rm{Polymer}}}}}}+{{{{{\rm{MDL}}}}}})}$$

To quantify the effectivity of loading the encapsulation efficiency was calculated:

2 $${{{{{\rm{encapsulation}}}}}}\; {{{{{\rm{efficiency}}}}}}=1-\frac{{m}({{{{{\rm{total}}}}}}\; {{{{{\rm{MDL}}}}}})-{m}({{{{{\rm{free}}}}}}\; {{{{{\rm{MDL}}}}}})}{{m}({{{{{\rm{total}}}}}}\; {{{{{\rm{MDL}}}}}})}$$

### Animals and cell culture

C57BL/6, B6.129S7-*Rag1*^*tm1Mom*^/J and C57BL/6-Tg(Foxp3-DTR/EGFP)23.2Spar/Mmjax (DEREG) mice were bread in the central animal facility of the University Medical Center Mainz (controlled 12/12–h light/dark cycle (lights on at 6:00 AM), temperature (22 ± 2 °C), and relative humidity (45–65%). All animal experiments were performed after approval by the Rhineland-Palatinate State Investigation Office (authorization G 17-1-069) in accordance with relevant laws, current institutional guidelines, and the Helsinki convention for the use and care of animals. The authors comply with the ARRIVE guidelines.

DEREG mice were depleted of Treg by intraperitoneal injection of 0.5 µg of diphtheria toxin (DT).

B16F10-OVA melanoma cells were grown at 37 °C in a humidified 5% CO_2_ atmosphere in an MDM culture medium containing 10% fetal bovine serum, 100 units/mL penicillin, 100 μg/ml streptomycin, and 500 µg/ml G418 (Geneticin). Cells were tested for mycoplasma every 3 months. B16F10-OVA melanoma cells (2 × 10^5^) were implanted by subcutaneous (s.c.) injection at an angle of 45° on the posterior flanks of 8–10 weeks old female mice. Tumor areas (length × width) were measured every second day using Vernier calipers and tumor volumes were calculated: Vol = (W*W*L*)/2. Mice were killed when there was visible necrosis or when the individual tumor size reached more than 2 cm in any direction.

For therapeutic treatment, micelles, MDL-12, or DMSO dissolved in buffer (PBS) were injected peritumorally using a 30-gauge needle syringe (Omnican 50, Braun Medical Inc, Melsungen, Germany). For this purpose, the skin at the tumor margin was lifted with forceps and the needle was inserted subcutaneously at a distance of 2 mm from the tumor periphery. A volume of 10 µl each was injected on the caudal and the rostral side.

### Cryogenic transmission-electron microscopy

5 µL of the micellar formulation solution (1 mg/mL, in MilliPore-water) were applied to freshly glow-discharged carbon grids with a copper 200 mesh (Quantfoil Micro Tools GmbH). Excess fluid was removed by direct blotting (2.5 s) and the grids were individually plunge-frozen in liquid ethane. Grids were cryotransferred in liquid nitrogen using a Gatan cryoholder (model 626 DH) to a Tecnai T12 transmission electron microscope equipped with a field emission electron source and operating at 120 kV accelerating voltage. Images were recorded using a TemCam-F416 (TVIPS, Gauting, Germany).

### Flow cytometry

Single-cell suspensions from blood, spleen, lymph nodes, liver, lung, and kidney were generated by passing through a 70 µm cell strainer, followed by erythrocyte lysis with ACK lysis buffer. Single-cell suspensions from tumors were generated by tissue digestion. Tumors were excised, cut into small pieces, and digested for 30 min at 37 °C in dissociation buffer (100 U/ml Collagenase Type II (Life Technologies), 100 μg/mL DNAse I (Roche) in RPMI 1640 + Glutamine supplemented with 10% FCS). The digested tumor suspension was strained using a 70 µm cell strainer and washed several times using MACS buffer (1 × PBS + 1 mM EDTA, 0.5% HSA). Cells were resuspended in FACS buffer (1 × PBS + 1 mM EDTA, 0.5% HSA and 20 μg/ml Sandoglobin). Flow cytometry data were acquired on an LSR II flow cytometer (BD Bioscience) with DIVA 8 and were later analyzed with FlowJo 10. For surface staining, cells were at first incubated with unlabeled mAb against CD16/CD32 in order to block nonspecific Fc receptor-mediated binding of staining antibodies. The cells were stained for 30 min at 4 °C with antibodies for the respective cell surface markers. Intranuclear staining was performed with a FoxP3 staining kit (eBioscience).

Based on a non-exclusive FSC/SSC gate singlets were gated by pulse geometry gating (SSC-A versus SSC-H) followed by dead cell exclusion and subsequent surface biomarker identification (Supplementary Fig. 4A).

### Confocal microscopy

B16 melanoma cells were injected s.c. on the posterior flank of B6 mice. Tumors were isolated 1 h after peritumoral injection of OG-labeled L-PM micelles and 4ʹ,6‐diamidino‐2‐phenylindole (DAPI, Molecular Probes, Ontario, Canada) at day 14 B16-OVA melanomas. Confocal images were taken on a high-speed spinning disc confocal microscope (Andor, Belfast, NI, UK) and DIC (CFI75) with an LWD 16× W water dipping series lens (Nikon).

### Dead cell determination

Isolated tumors (2.5 × 10^6^ cells) were surface-stained and suspended in 100 µL 1× Annexin binding buffer (Invitrogen). 5 µl of PE-conjugated annexin V and 5 µl of 7-AAD (7-Amino-actinomycin; Sigma A-9400) were added and tubes were gently vortexed followed by 15 min incubation at RT in the dark. After the addition of 900 µL, 1× binding buffer cells were analyzed within 1 h of staining on a BD LSRII (RBV configuration).

### Antibodies

The following primary antibody clones were used for flow cytometry analyses: anti-CD16/CD32 (clone 93; eBioscience, #14-0161-82, used at 1:50), anti-CD45PAN-BV711 (clone 30-F11; BioLegend, #103147, used at 1:100), anti-CD45PAN-APC-eFluor 780 (clone 30-F11; eBioscience, #47-0451-82, used at 1:60), anti-CD11b-PE (clone M1/70; BD Biosciences, #557397, used at 1:100), anti-CD29-Alexa Fluor 488 (clone HMß1-1; BioLegend, #102212, used at 1:80), Fixable Viability Dye (eFluor 506; eBioscience) was used for dead cell exclusion.

### Determination of intracellular cAMP levels by ELISA

Intracellular cAMP concentrations were measured using a Cyclic AMP EIA kit (Cayman Chemical). 1 × 10^6^ cultured cells were lysed in lysing buffer (150 mM NaCl, 50 mM Tris–HCl (pH 7,6), 5 mM EDTA, 1,5 mM MgCl_2_, 1% Triton X, 5% Glycerol, 50 mM Na-ß-glycerophosphate, 20 mM Na-Pyrophosphat, 10 mM NaF, 10 mM Na_2_HPO_4_ supplemented with inhibitors 1 mM Na_3_VO_4_ and 100 mM Complete, Mini EDTA-free) prior to cAMP determination. To determine cAMP levels in tumor tissue, 10 mg tissue pieces (edge to center) were separated on a scale and immediately frozen in liquid nitrogen. Snap frozen tissue was ground to a fine powder under liquid nitrogen with stainless steel pestle and mortar. The powder was homogenized in 100 µL lysing buffer and stored at −80 until cAMP analysis. The cAMP ELISA was performed according to the manufacturer’s instructions. Optical densities were determined on a HidexSense Beta microplate reader.

### Whole transcriptome single-cell RNA-sequencing

B16 melanoma cells were injected s.c. on the posterior flank of B6 mice (see the section “Animals and cell culture”) and tumors were treated with L-PM and as control with NL-PM (see the section “Melanoma growth inhibition with MDL-loaded polypept(o)ide micelles”). At day 15 single-cell suspensions from NL-PM and L-PM treated tumors (*n* = 3) were generated by tissue digestion using murine tumor dissociation Kit and gentleMACS^TM^ dissociator (Miltenyi Biotec). Tumor-infiltrating CD45^+^ immune cells were enriched by murine CD45 (TIL) MicroBeads (Miltenyi Biotec) using MACS technology. Cells were washed with FACS buffer (PBS + 0.5% BSA + 5 mM EDTA) and stained with fixable viability dye (eBioscience) and CD45 antibody (eBioscience) for 30 min at 4 °C. To enrich viable CD45^+^ cells were sorted using an Aria III flow cytometer (BD Bioscience). The purity of sorted cells was >95%.

Single cells were counted and captured using the BD Rhapsody Single-Cell Analysis System following the manufacturer’s guidelines (BD Biosciences). Whole transcriptome analysis (WTA) library prep and Sample Tag library prep were generated following the BD Rhapsody System mRNA WTA and Sample Tag Library Preparation Protocol (BD Biosciences). Samples were sequenced using the Illumina NovaSeq 6000 Sequencing System (Novogene, Cambridge, UK) and a 150PE strategy. The resulting raw data were preprocessed according to the Illumina standard protocol and transcript alignment, counting, and demultiplexing were performed using the BD Rhapsody WTA analysis pipeline yielding 4388 called putative cells overall with a mean of 160,000 aligned reads per cell. Pipeline output RSEC Mols per Cell files were imported in Partek Flow software (Version 9.0) and cells that contained <500 detected genes and a percentage of mitochondrial counts higher than 15 were removed from subsequent analysis. Single-cell counts were normalized by Partek Flows recommended normalization order (CPM (counts_per_million), Add:1 and log2). For dimensionality reduction PCA was used, followed by graph-based clustering (using the SmartLocalMoving (SLM) clustering algorithm) and t-SNE algorithm for visualization. Populations shown in 2D t-SNE projections were annotated according to ImmGen’s (www.immgen.org) data browser. To compare an equal number of total cells in NL-PM- and L-PM- treated samples, cell counts were randomly downsampled (leading to 2820 cells in total, 1410 cells per each group NL-PM and L-PM). To study anti-inflammatory signature (gene set comprises *Ptgs2*, *Vegfa*, *Egfr*, *Arg1*, *Ccl22*, *Ccl17*, *Il10*, *Il12a*) in macrophages and neutrophils, AUCell analysis was performed in Partek Flow (Aibar et al. (2016) AUCell: Analysis of ‘gene set’ activity in single-cell RNA-seq data). To define exhausted T cells, expression of *Pdcd1(PD-1*), *Havcr2 (TIM3*), *Ctla4*, *Tigit,* and *Lag3* was analyzed in both groups) and visualized by a heatmap after hierarchical clustering (cluster distance metric = average linkage; point distance metric = Euclidean) and a 2D scatter plot.

### Statistics and reproducibility

Unless otherwise indicated, all experiments underlying the figures and supplementary figures were repeated independently at least three times with similar results. All measurements were taken from distinct samples. Statistical evaluation was performed using GraphPad Prism version 8.0.2. Normality was assessed for all data sets (*n* < 50) with the Shapiro–Wilk to decide for nonparametric (Figs. 1C and 4B, C) or parametric (all other datasets) testing. Parametric testing of two groups was performed using an unpaired two-sided *t*-Test with 95% confidence interval, 0.05 significance level (alpha) and Holm–Šídák corrected *P*-values. For more than two groups, an ordinary one-way ANOVA with Holm–Šídák correction of multiple comparisons was chosen.

Data-rich collections from scRNA Seq experiments were cleared for outliers with the ROUT function (*Q* set to 1%) using Prism 8.0.2. and the selection was confirmed by observing the PCA-Plot for principal components 1 and 2 using PartekFlow 9.0 (check source data for excluded values). As expected, scRNA-Seq data did not follow a normal distribution as tests for (log) normal distribution were not passed (Shapiro–Wilk/D´Agostino–Pearson) Therefore an unpaired non-parametric two-sided Mann-Whitney test was performed. Differences were considered significant according to *NJEM* style (ns ≤ 0.12, *P ≤ 0.033, **P ≤ 0.002, ***P ≤ 0.001).

### Reporting summary

Further information on research design is available in the Nature Research Reporting Summary linked to this article.

## Supplementary information

Supplementary Information

Description of Additional Supplementary Files

Supplementary Movie 1

Reporting Summary

## Data Availability

In addition to the data available in the article and supplementary information, a Source Data underlying all figures and supplementary figures generated in this study have been deposited in the Figshare database [10.6084/m9.figshare.16419204.v1]. Single-cell RNA-Seq data from this project have been deposited within the NCBI Gene Expression Omnibus (GEO) database under accession number: GSE166028.
