# Supplementary material for: Therapeutic melanoma inhibition by local micelle-mediated cyclic nucleotide repression
Source: Nat Commun. 2021 Oct 13;12:5981. doi: 10.1038/s41467-021-26269-w (PMC8514514; doi:10.1038/s41467-021-26269-w)
Supplement: Supplementary file 1 — Supplementary Information [file 41467_2021_26269_MOESM1_ESM.pdf]

Johann et al., **Therapeutic melanoma inhibition by local micelle-mediated cyclic nucleotide repression**

Supplementary Information

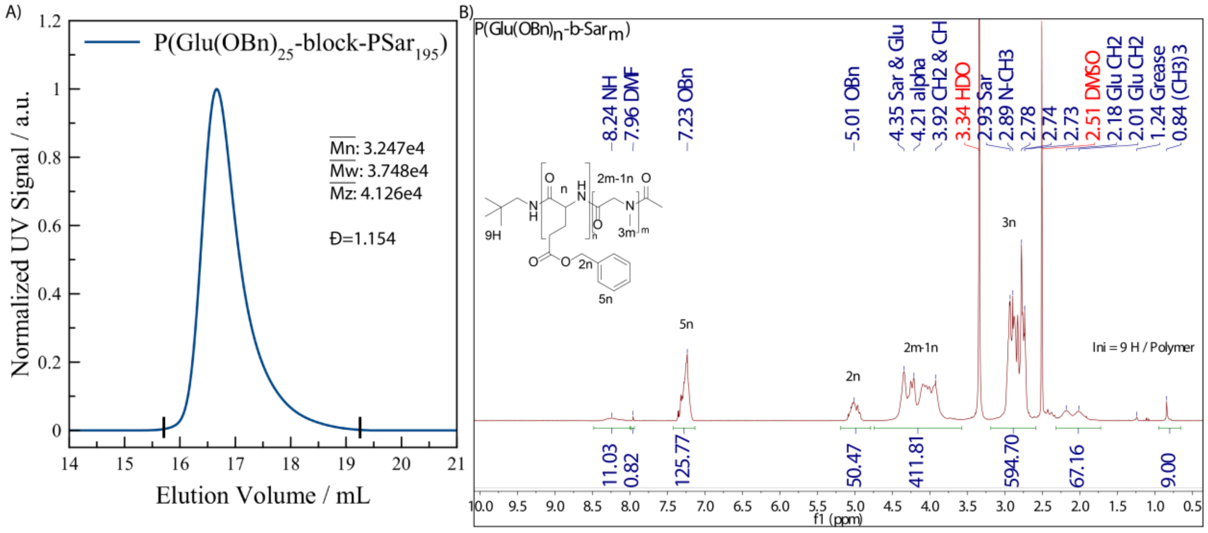

Supplementary Figure 1: Characterization of pGlu(OBn)-*b*-pSar. (A) Elution profile and core GPC analysis data for pGlu(OBn)$_{25}$-*b*-pSar$_{195}$ with black bars indicating integration borders. (B) 400 MHz ^{1}H NMR in DMSO-d_6 of utilized block copolymers for aggregation, marked signals were used for end group analysis by comparison with 9H of the initiator.

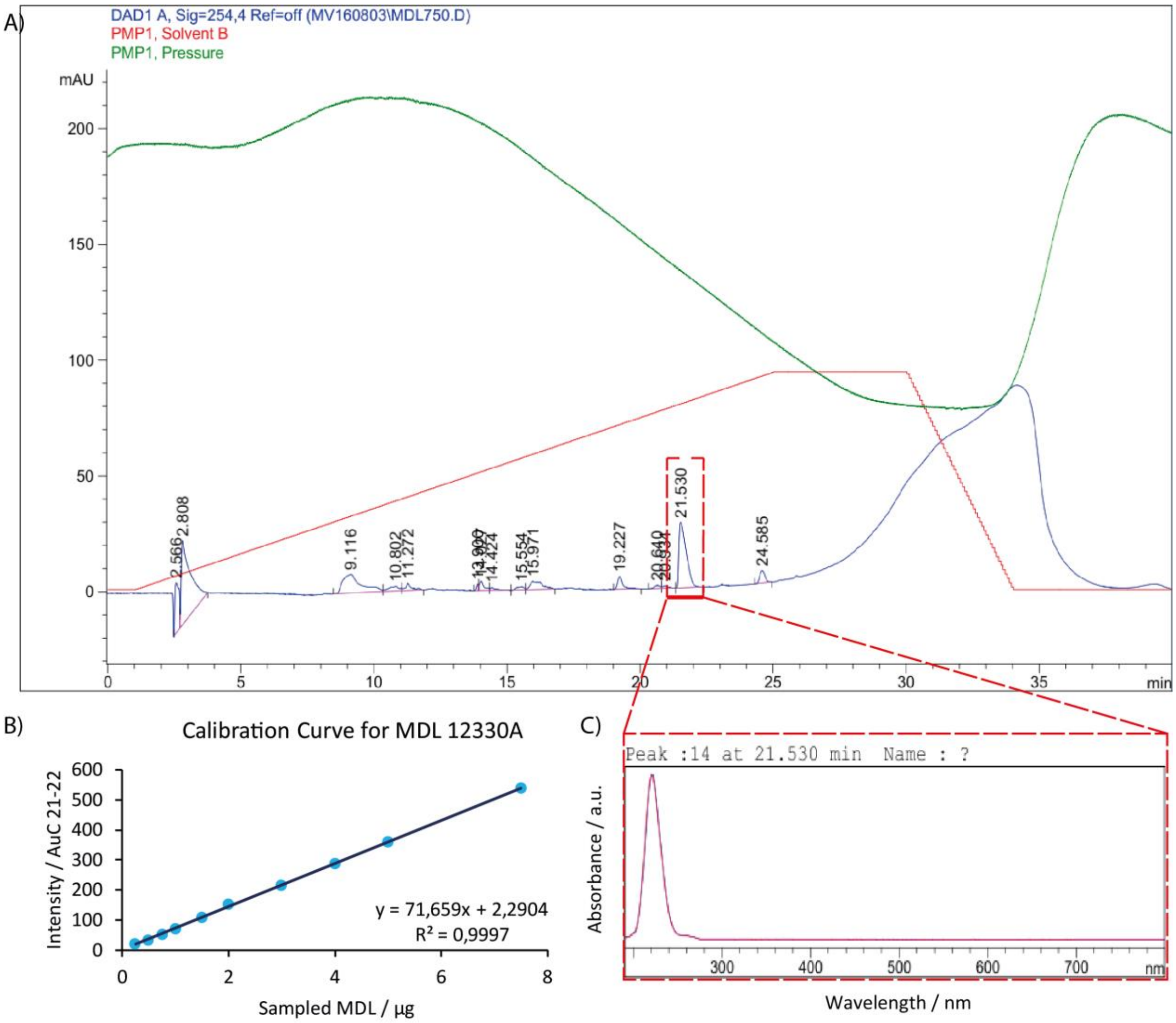

Supplementary Figure 2: HPLC Calibration. (A) Original HPLC elugram and (B) obtained calibration curve showing the linear concentration to AUC relation. (C) The absorption spectrum recorded by the photodiode array detector was enlarged to be compared with the sample peak besides elution time.

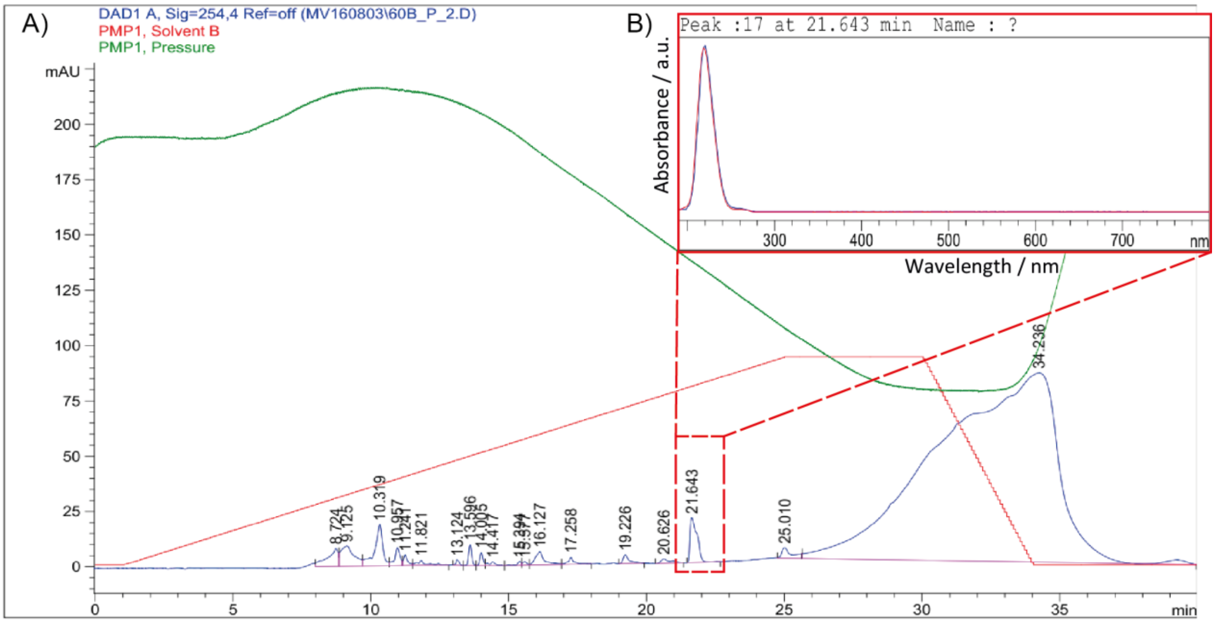

Supplementary Figure 3. Sample quantification. (A) Example HPLC elugram of sample for quantification. (B) MDL peak highlighted and UV-Vis photodiode array spectrum of peak recorded for comparison.

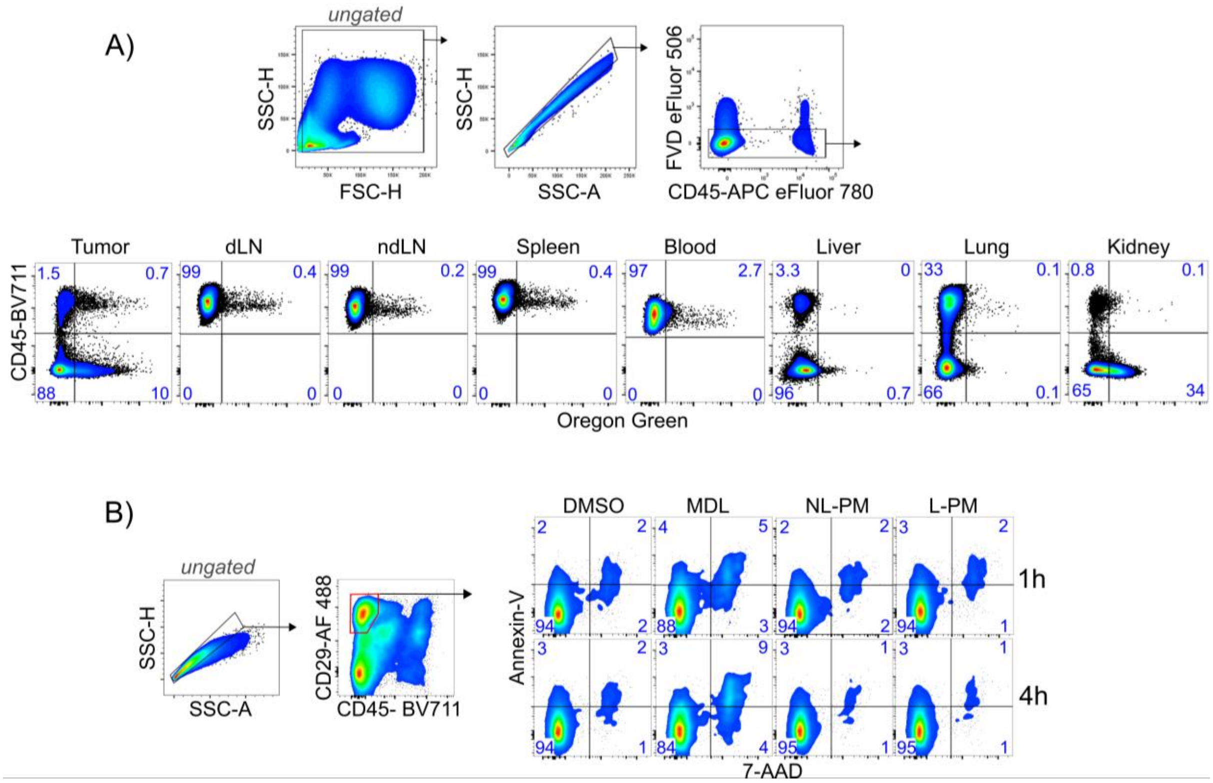

Supplementary Figure 4: Body distribution and melanoma cell death upon peritumoral micelle injection. (A) Upper row: Gating by scatter and dead cell exclusion, FVD= Fixable Viability Dye. Lower row: Representative flow cytometric plots of fluorescent cells in the (d14) tumor, tumor-draining lymph nodes (dLN), non-draining lymph nodes (ndLN), spleen, blood, liver, lung and kidney of B6 mice 24h after peritumoral injection with Oregon-green labelled micelles. Numbers represent frequencies in gates. Complete data summarized in Fig. 2B.

(B) Representative flow cytometric analysis of dead tumor cells in day 14 melanoma. Representative flow cytometric Annexin-V/7-AAD plots of scatter pre-gated and $CD45^{neg}$ $CD29^{+}$ gated B16 melanoma cells 1h and 4h after peritumoral injection of MDL-12 (10 µM) or L-PM (10 µM MDL), NL-PM or DMSO. Numbers represent frequencies in gates. Complete data summarized in Fig. 2C.

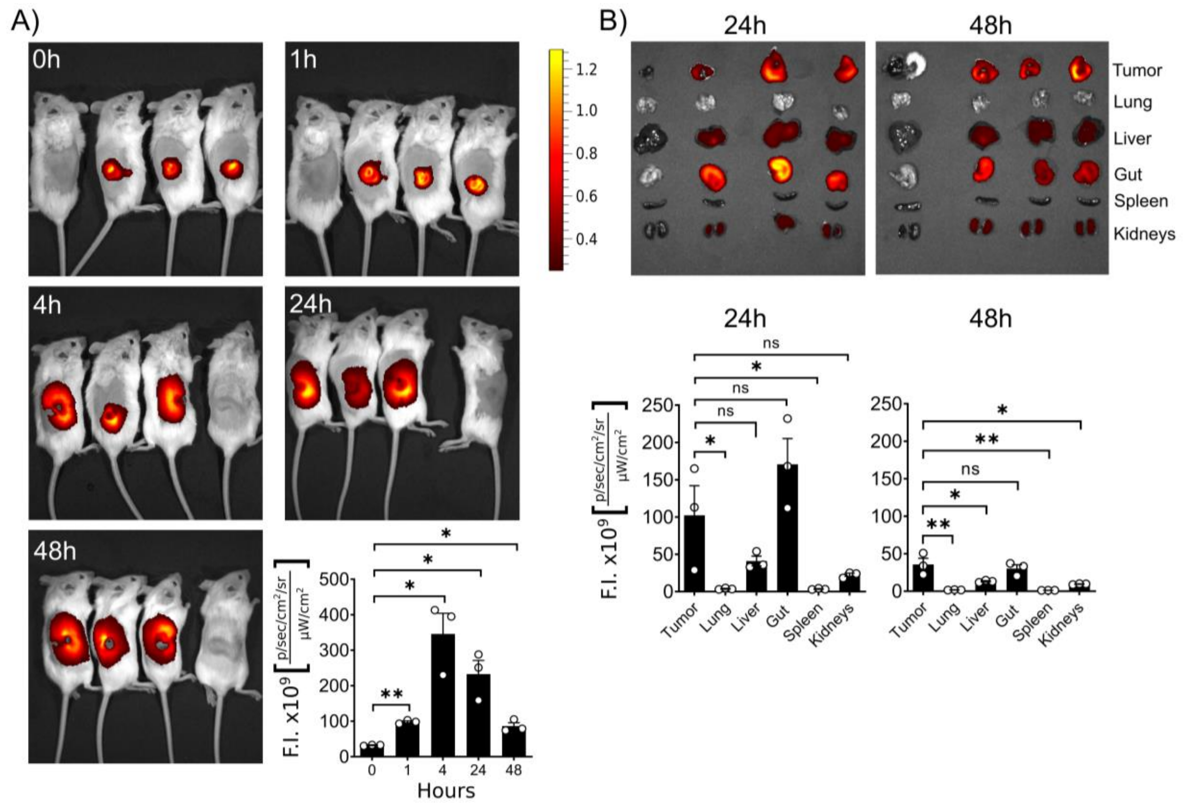

Supplementary Figure 5: In vivo-distribution of peritumorally injected Indocyanine Green (ICG)-loaded micelles. (A) B16F10-OVA melanoma-bearing C57BL/6 albino mice at day 14 after tumor inoculation received a peritumoral injection of ICG-loaded micelles and epifluorescence was imaged at indicated time points using the IVIS image system. Individual non-injected animals on the left or right. **P=0,0023 (1h), *P=0,03 (4h), *P=0,0353 (24h), *P=0,03 (48h) (B) Tissue distribution analysis at 24 and 48 hours, n=3 micelle-injected animals + n=1 non-injected control animal per time point/group. Data presented as mean ± SEM of micelle-injected animals. At 24 hrs: *P=0.03 (T vs Lu), *P=0.03 (T vs Sp). At 48 hrs: **P=0.0074 (T vs Lu), *P=0,0269 (T vs Li), **P=0.007 (T vs Sp) *P=0.0171 (T vs Ki), ns = non significant (unpaired t-test, two-sided, with Holm-Šídák-corrected P values, alpha = 0.05, 0.95 confidence interval). Controls not included in quantification.

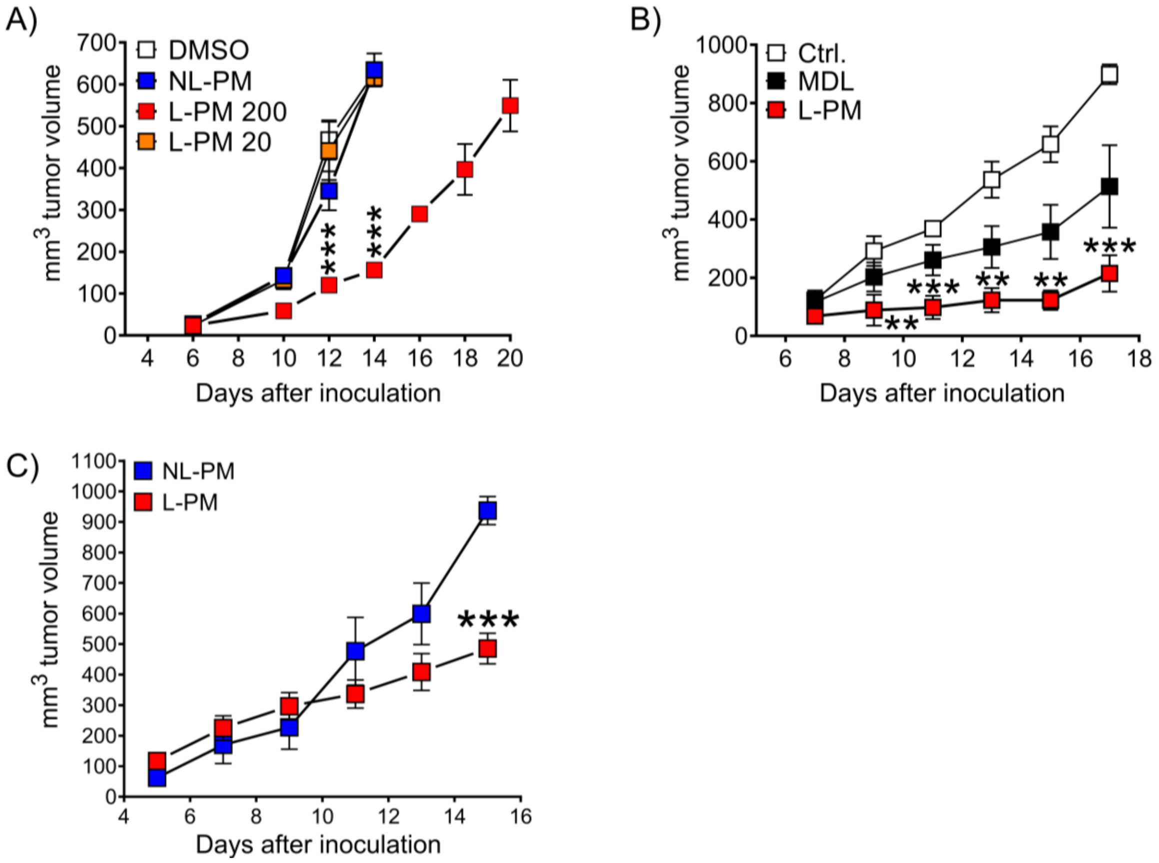

Supplementary Figure 6: Dose-dependency, micelle-dependency and time-dependency of melanoma inhibition. (A) Dose-dependency of micelle-mediated melanoma inhibition. C57BL/6 mice were subcutaneously inoculated with B16-OVA tumor cells and received peritumoral micelle injections with 200 or 20 µM (L-PM 200 and L-PM 20) total drug content every other day starting from day 4 after inoculation. Representative results of three independent experiments shown as mean ± SEM, DMSO (white) *n* = 5, NL-PM (blue) *n* = 5 and L-PM 200 (red) *n* = 5, L-PM 20 (orange) *n* = 4, NL-PM vs L-PM 200 ***P=0.00098 (day 12), ***P=0.001 (day 14) (parametric unpaired *t*-test, two-sided, Holm-Šídák corrected for multiple comparisons, alpha 0.05, confidence interval 0.95). (B) Melanoma growth suppression of MDL-loaded micelles compared to the free drug. C57BL/6 mice were subcutaneously inoculated with B16-OVA tumor cells and received peritumoral micelle or MDL injections (200 µM, black) every other day starting from day 4 after inoculation. Representative results of two independent experiments shown as mean ± SEM, individual tumors shown, Ctrl. (untreated) *n* = 3 and MDL *n* = 3, L-PM *n* = 6. Ctrl vs L-PM *P=0.01 (day 9), ***P=0.0009 (day 11), **P=0.006 (day 13), **P=0.005 (day 15), ***P=0.0009 (day 17) (parametric unpaired *t*-test, two-sided, Holm-Šídák corrected for multiple comparisons, alpha 0.05, confidence interval 0.95). (C) Growth suppression of B16 melanoma cells by delayed treatment. B16-inoculated C57BL/6 mice received peritumoral micelle injections every other day. L-PM injection was postponed to day 6 after inoculation. Representative results of two independent experiments shown as mean ± SEM, *n* = 5 per group. NL-PM vs L-PM: ***P=0.000998 (day 15) (parametric unpaired *t*-test,

two-sided, Holm-Šídák corrected for multiple comparisons, alpha 0.05, confidence interval 0.95).

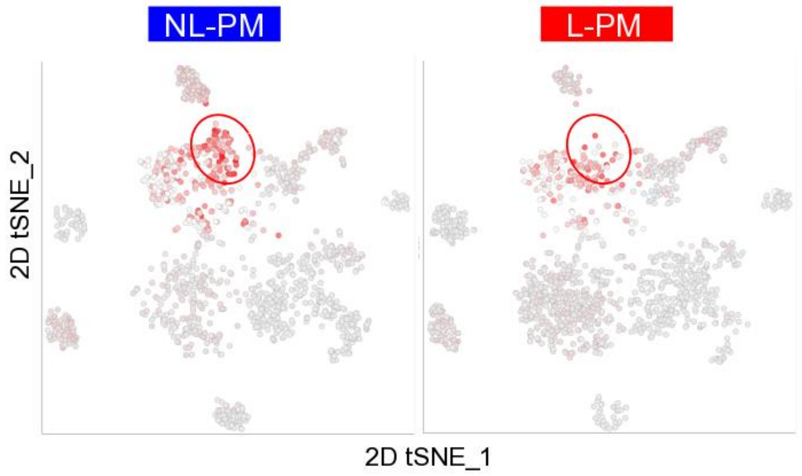

Supplementary Figure 7: Micellar peritumoral cAMP repression reduces the number of anti-inflammatory monocytes/macrophages in the tumor. 2D tSNEs of immune cells in NL-PM and L-PM-treated B16-OVA melanomas. Monocytes/macrophages with anti-inflammatory signature shown in red. Source data are provided as a Source Data file. Single-cell RNA-Seq data accessible via NCBI Gene Expression Omnibus (GEO) database accession number: GSE166028 [https://www.ncbi.nlm.nih.gov/geo/query/acc.cgi?acc=GSE166028].
