# Supplementary material for: Therapeutic melanoma inhibition by local micelle-mediated cyclic nucleotide repression
Source: Nat Commun. 2021 Oct 13;12:5981. doi: 10.1038/s41467-021-26269-w (PMC8514514; doi:10.1038/s41467-021-26269-w)
Supplement: Supplementary file 2 — Description of Additional Supplementary Files [file 41467_2021_26269_MOESM2_ESM.docx]

**Description of Additional Supplementary Files**

File Name: Supplementary Movie 1

Description:
Z-stack of confocal microscopic images of the margin of a B16 tumor, 1 hour after peritumoral injection of Oregon Green (OG)-labeled micelles. B16-OVA melanoma cells were injected s.c. on the posterior flank of 8-10 weeks old female C57BL/6 mice. 14 days after inoculation, tumors were peritumorally injected with OG-labeled L-PM micelles and 4ʹ, 6‐diamidino‐2‐phenylindole (DAPI, Molecular Probes, Ontario, Canada). Confocal images were taken on a high-speed spinning disc confocal microscope (Andor, Belfast, NI, UK) and DIC (CFI75) with a LWD 16x W water dipping series lens (Nikon). Images representative of n = 3 independent experiments.
