# Supplementary material for: Therapeutic melanoma inhibition by local micelle-mediated cyclic nucleotide repression
Source: Nat Commun. 2021 Oct 13;12:5981. doi: 10.1038/s41467-021-26269-w (PMC8514514; doi:10.1038/s41467-021-26269-w)
Supplement: Supplementary file 4 — Reporting Summary [file 41467_2021_26269_MOESM4_ESM.pdf]

nature research

Corresponding author(s): Matthias Barz, Tobias Bopp, Christian Becker

Last updated by author(s): Christian Becker, Verena K Raker

Reporting Summary

Nature Research wishes to improve the reproducibility of the work that we publish. This form provides structure for consistency and transparency in reporting. For further information on Nature Research policies, see our Editorial Policies and the Editorial Policy Checklist.

Statistics

For all statistical analyses, confirm that the following items are present in the figure legend, table legend, main text, or Methods section.

n/a	Confirmed	
☐	☒	The exact sample size (*n*) for each experimental group/condition, given as a discrete number and unit of measurement
☐	☒	A statement on whether measurements were taken from distinct samples or whether the same sample was measured repeatedly
☐	☒	The statistical test(s) used AND whether they are one- or two-sided<br>*Only common tests should be described solely by name; describe more complex techniques in the Methods section.*
☐	☒	A description of all covariates tested
☐	☒	A description of any assumptions or corrections, such as tests of normality and adjustment for multiple comparisons
☐	☒	A full description of the statistical parameters including central tendency (e.g. means) or other basic estimates (e.g. regression coefficient) AND variation (e.g. standard deviation) or associated estimates of uncertainty (e.g. confidence intervals)
☐	☒	For null hypothesis testing, the test statistic (e.g. *F*, *t*, *r*) with confidence intervals, effect sizes, degrees of freedom and *P* value noted<br>*Give P values as exact values whenever suitable.*
☒	☐	For Bayesian analysis, information on the choice of priors and Markov chain Monte Carlo settings
☒	☐	For hierarchical and complex designs, identification of the appropriate level for tests and full reporting of outcomes
☒	☐	Estimates of effect sizes (e.g. Cohen's *d*, Pearson's *r*), indicating how they were calculated

Our web collection on statistics for biologists contains articles on many of the points above.

Software and code

Policy information about availability of computer code

Data collection	MestReNova 10.0, DIVA 8,
Data analysis	MestReNova 10.0, WinGPC UniChrome 8.00 (Build 994), Spectra Manager Version 2.0, FlowJo V10.07, Partek Flow (Version 9.0), GraphPad Prism version 8.0.2

For manuscripts utilizing custom algorithms or software that are central to the research but not yet described in published literature, software must be made available to editors and reviewers. We strongly encourage code deposition in a community repository (e.g. GitHub). See the Nature Research guidelines for submitting code & software for further information.

Data

Policy information about availability of data

All manuscripts must include a data availability statement. This statement should provide the following information, where applicable:

- Accession codes, unique identifiers, or web links for publicly available datasets
- A list of figures that have associated raw data
- A description of any restrictions on data availability

Single-cell RNA-Seq data from this project have been deposited within the NCBI Gene Expression Omnibus (GEO) database under accession number: GSE166028. The source data underlying all figures and supplementary figures are available via Figshare https://doi.org/10.6084/m9.figshare.15262728.v1.

Field-specific reporting

Please select the one below that is the best fit for your research. If you are not sure, read the appropriate sections before making your selection.

- [x] Life sciences
- [ ] Behavioural & social sciences
- [ ] Ecological, evolutionary & environmental sciences

For a reference copy of the document with all sections, see nature.com/documents/nr-reporting-summary-flat.pdf

Life sciences study design

All studies must disclose on these points even when the disclosure is negative.

Sample size	Sample sizes for cell culture experiments were determined according to pilot studies or previous experimental experience and standard protocols in the field (Bohn. et al., 2018, Nat Imm, Dal et al., 2020, Small)<br>Animal numbers were calculated according to Guidelines for the Care and Use of Mammals in Neuroscience and Behavioral Research 2003, ISBN: 0-309-50587-9 and approved in a study protocol by the State Investigation Office Rhineland-Palatinate.<br>The sample calculation for RNA-Seq is based on a calculation by a statistician of the Institute of Medical Biometry (IMBEI) Mainz and published power analyses (J Comput Biol. 2012;20:970-978, RNA. 2014;20:1684-1696).
Data exclusions	No data was excluded
Replication	Unless otherwise indicated, all experiments underlying the figures and supplementary figures were repeated independently at least three times. All attempts at replication were successful.<br><br>The single cell RNA sequencing approach was performed with cells isolated from a total of 6 individual mice resulting in an average of app. 4300 CD45+ cells and a mean of 160.000 aligned reads per cell. Due to the very clear-cut and representative results across all biological replicates within one group, additional experiments were not conducted to avoid possible and unwanted batch effects due to technical issues.<br><br>To analyze the in vivo distribution of the micelles, three different methodological approaches (flow cytometry and confocal microscopy using OregonGreen (OG)-labelled micelles, IVIS measurements using Indocyanine Green (ICG) loaded micelles) were used. Due to the comparable and unambiguous results obtained by both flow cytometry and confocal microscopy, no further replicates were conducted for ICG epifluorescence measurement.
Randomization	Allocation was random
Blinding	Investigators were blinded

Reporting for specific materials, systems and methods

We require information from authors about some types of materials, experimental systems and methods used in many studies. Here, indicate whether each material, system or method listed is relevant to your study. If you are not sure if a list item applies to your research, read the appropriate section before selecting a response.

Materials & experimental systems

n/a	Involved in the study	
[ ]	[x]	Antibodies
[ ]	[x]	Eukaryotic cell lines
[x]	[ ]	Palaeontology and archaeology
[ ]	[x]	Animals and other organisms
[x]	[ ]	Human research participants
[x]	[ ]	Clinical data
[x]	[ ]	Dual use research of concern

Methods

n/a	Involved in the study	
[x]	[ ]	ChIP-seq
[ ]	[x]	Flow cytometry
[x]	[ ]	MRI-based neuroimaging

Antibodies

Antibodies used	anti-CD16/CD32 (clone 93; eBioscience, #14-0161-82, used at 1:50)<br>anti-CD45PAN-BV711 (clone 30-F11; BioLegend, #103147, used at 1:100)<br>anti-CD45PAN-APC-eFluor 780 (clone 30-F11; eBioscience, #47-0451-82, used at 1:60)<br>anti-CD11b-PE (clone M1/70; BD Biosciences, #557397, used at 1:100)<br>anti-CD29-Alexa Fluor 488 (clone eBioHMßb1-1; BioLegend, #102212, used at 1:80),
Validation	All antibodies used here are commercially available and have validated for the species and application. Validation Information as well as references can be found for each individual antibody in the data sheets provided by the companies.

Eukaryotic cell lines

Policy information about cell lines

Cell line source(s)	OVA expressing B16F10 (B16) melanoma cells were provided by M. Diken (TrOn, University Medical Center Mainz).
Authentication	B16F10-OVA cells were not authenticated.
Mycoplasma contamination	All cell lines tested negative for mycoplasma contamination.
Commonly misidentified lines (See ICLAC register)	No commonly misidentified cell lines were used in the study.

Animals and other organisms

Policy information about studies involving animals; ARRIVE guidelines recommended for reporting animal research

Laboratory animals	Mus musculus, 8-10 weeks old female C57BL/6, B6.129S7-Rag1tm1Mom/J and C57BL/6-Tg(Foxp3-DTR/EGFP)23.2Spar/Mmjax. (DEREG) mice
Wild animals	No wild animals have been used
Field-collected samples	No field collected samples have been used
Ethics oversight	All animal experiments were performed under a study protocol approved by the Rhineland-Palatinate State Investigation Office (approval number G 17-1-069) in accordance with relevant laws, applicable institutional guidelines, and the Helsinki Convention on the Use and Care of Animals. The authors adhere to the ARRIVE guidelines.

Note that full information on the approval of the study protocol must also be provided in the manuscript.

Flow Cytometry

Plots

Confirm that:

- [x] The axis labels state the marker and fluorochrome used (e.g. CD4-FITC).
- [x] The axis scales are clearly visible. Include numbers along axes only for bottom left plot of group (a 'group' is an analysis of identical markers).
- [x] All plots are contour plots with outliers or pseudocolor plots.
- [x] A numerical value for number of cells or percentage (with statistics) is provided.

Methodology

Sample preparation	Single cell suspensions from blood, spleen, lymph nodes, liver, lung and kidney were generated by passing through a 70 µm cell strainer, followed by erythrocyte lysis with ACK lysis buffer. Single cell suspensions from tumors were generated by tissue digestion. Tumors were excised, cut into small pieces and digested for 30 min at 37 °C in dissociation buffer (100 U/ml Collagenase Type II (Life Technologies), 100 µg/mL DNAse I (Roche) in RPMI 1640 + Glutamine supplemented with 10% FCS). The digested tumor suspension was strained using a 70 µm cell strainer and washed several times using MACS buffer (1x PBS + 1 mM EDTA, 0.5% HSA). Cells were resuspended in FACS buffer (1x PBS + 1 mM EDTA, 0.5% HSA and 20 µg/ml Sandoglobin).
Instrument	BD LSRII (RBV configuration)
Software	data collection: DIVA 8<br>data analysis: FlowJo V10.7
Cell population abundance	Purity of sorted cells was >95% (whole Transcriptome single-cell RNA-sequencing)
Gating strategy	Based on a non-exclusive FSC/SSC gate singlets were gated by pulse geometry gating (SSC-A versus SSC-H) followed by dead cell exclusion and subsequent surface biomarker identification (Supplemental Figure 4A).

- [x] Tick this box to confirm that a figure exemplifying the gating strategy is provided in the Supplementary Information.
